# Analysis of the Nucleophilic Solvation Effects in Isopropyl Chlorothioformate Solvolysis

**DOI:** 10.3390/ijms11072597

**Published:** 2010-06-29

**Authors:** Malcolm J. D’Souza, Brian P. Mahon, Dennis N. Kevill

**Affiliations:** 1 Department of Chemistry, Wesley College, 120 N. State Street, Dover, DE 19901-3875, USA; 2 Department of Chemistry and Biochemistry, Northern Illinois University, DeKalb, IL 60115-2862, USA

**Keywords:** solvolysis, Grunwald-Winstein Equations, nucleophilic solvation, chlorothioformate

## Abstract

Correlation of the solvent effects through application of the extended Grunwald-Winstein equation to the solvolysis of isopropyl chlorothioformate results in a sensitivity value of 0.38 towards changes in solvent nucleophilicity (*l*) and a sensitivity value of 0.72 towards changes in solvent ionizing power (*m*). This tangible *l* value coupled with the negative entropies of activation observed indicates a favorable predisposition towards a modest rear-side nucleophilic solvation of a developing carbocation. Only in 100% ethanol was the bimolecular pathway dominant. These observations are very different from those obtained for the solvolysis of isopropyl chloroformate, where dual reaction channels were proposed, with the addition-elimination reaction favored in the more nucleophilic solvents and a unimolecular fragmentation-ionization mechanism favored in the highly ionizing solvents.

## 1. Introduction

The application [1] of the simple [2] and extended [3] (two-term) Grunwald-Winstein Equations for the analyses of the specific rates of solvolyses of chloroformates [4–24], and their corresponding sulfur-for-oxygen substituted analogs (thiono-, thio-, and dithiochloroformate esters) [1,10,21,25–30] has proven to be immensely beneficial [1] for the consideration of solvent effects upon reactions of these important intermediates that are found in pharmaceutical [31] and agricultural [32] products, and as derivatization agents in the flavor and fragrance industries [33]. In the simple [2] (Equation 1) and extended [3] (Equation 2) Grunwald-Winstein Equations, *k* and *k_o_* are the specific rates of solvolysis in a given solvent and in the standard solvent (80% ethanol), respectively, *m* represents the sensitivity to changes in the solvent ionizing power *Y* (initially set at unity for *tert*-butyl chloride solvolyses), and *c* is a constant (residual) term. In Equation 2, the additional *lN* term was added to include a term governed by the sensitivity *l* to changes in solvent nucleophilicity (*N*). When using Equation 1 for ionization (S_N_1 + E1) mechanisms, it was realized both that the ionizing scales are leaving-group dependent and that adamantyl derivatives provide better standard substrates, and now for each commonly used leaving group X a series of *Y_X_* values [34–36] are available. For bimolecular (S_N_2 and/or E2) reactions, *N_T_* scales based on the solvolyses of the *S*-methyldibenzothiophenium ion [37,38] have become the recognized standards for considerations of solvent nucleophilicity. To correct for dispersions in Grunwald-Winstein plots observed whenever the reaction center is adjacent to a π-system [39,40], or in α-haloalkyl aryl compounds that proceed via anchimeric assistance (*k*_Δ_) [41], Kevill and D’Souza proposed [1,42–45] the addition of an additional aromatic ring parameter (*hI*) term to Equations 1 and 2 to give Equations 3 and 4. In Equations 3 and 4, *h* represents the sensitivity of solvolyses to changes in the aromatic ring parameter *I*. Recently, it was stressed [45] that the additional *hI* term can only be applied in situations where there is the presence of conjugated π-electrons adjacent to the developing carbocationic center, or in substrates that proceed with charge delocalization via a 1,2-aryl shift.

(1) $$\text{log}\left(k/k_{o}\right)=mY+c$$

(2) $$\text{log}(k/k_{o})=lN+mY+c$$

(3) $$\text{log}(k/k_{o})=mY+hI+c$$

(4) $$\text{log}(k/k_{o})=lN+mY+hI+c$$

**Figure 1.**
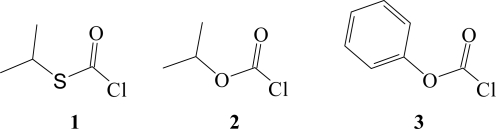
Molecular structures of *s*-isopropyl chlorothioformate (**1**), *s*-isopropyl chloroformate (**2**), and *s*-phenyl chloroformate (**3**).

After surveying the literature [29,46–54] it was pointed out [29,31], that the most stable geometries for RXCXCl (where X = S or O) exist in a configuration where the C=O or the C=S is *syn* with respect to R. Furthermore, studies [55] based on high resolution microwave spectra have shown that the *s*-isopropyl thioesters have a higher torsional barrier when compared to the *s*-ethyl thioesters. In Figure 1, the molecular structures for *s*-isopropyl chlorothioformate (**1**), *s*-isopropyl chloroformate (**2**), and *s-*phenyl chloroformate (**3**) are shown where the halogen atom is in a *trans* position with respect to the alkyl or aryl group *i.e.*, the structures are drawn in a *syn* conformation.

**Scheme 1.**
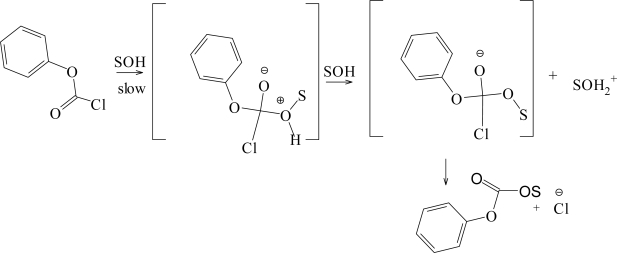
Stepwise addition-elimination mechanism through a tetrahedral intermediate for phenyl chloroformate (**3**).

The solvolysis of phenyl chloroformate (PhOCOCl, **3**) depicted in Scheme 1 has been extensively studied [7,21] over the full range of the types of solvent usually incorporated into studies utilizing the extended Grunwald-Winstein equation (Equation 2). The observed sensitivity values of *l* = 1.66 and *m* = 0.56 are now taken as typical values [1,7,10,12–16,18,20–28,30,43] for attack at an acyl carbon proceeding by the addition-elimination mechanism, with the addition step being rate-determining. A sulfur-for-oxygen substitution for both oxygen atoms in **3** yields the dithio analog, phenyl dithiochloroformate (PhSCSCl). Recent application [21,25] of Equations 2 and 4 to the solvolysis of PhSCSCl generated large *l* values of 0.69, and 0.80, respectively, and *m* values of 0.95 using Equation 2, and 1.02 using 4. The appreciable *l* values signify a profound rear-side nucleophilic solvation occurring at the developing resonance stabilized carbocation. Also, the *h* value of 0.42 ± 0.15 obtained [21] using Equation 4 suggests a minimal charge delocalization into the aromatic ring. The substitution of the ether oxygen of **3** with sulfur yields phenyl chlorothioformate (PhSCOCl), and the substitution of the carbonyl oxygen of **3** with sulfur, produces phenyl chlorothionoformate (PhOCSCl). It was shown [21,25,27,28,56] that PhSCOCl and PhOCSCl solvolyze with very similar specific rates of solvolysis by dual mechanisms, that have an essential identical division [25] into the addition-elimination (A–E) and ionization (S_N_1) pathways as the solvent is varied. This suggests that the inductive effect of the phenoxy group in PhOCSCl is counterbalanced by the conjugative release of the thiocarbonyl group. The reverse occurs in PhSCOCl, where the mesomeric effect of the thiophenoxy substituent is redressed by the electron-withdrawing character of the carbonyl group.

On the other hand, superimposed mechanisms [1,10,12,13,16,18–21,24,30,57–62] were observed for the alkyl chloroformate (ROCOCl) and chlorothioformate (RSCOCl) esters and the ranges of dominance for each were found to be heavily dependent on the nature of the substituent (R), solvent nucleophilicity, and on the ionizing ability of the solvent. These solvolyses could be treated, by application of Equation 2, in terms of two correlations with very different sets of *l* and *m* values, believed to correspond to the side-by-side operation of the bimolecular addition-elimination and unimolecular ionization (S_N_1) pathways. Queen’s original proposal [58–60] for the hydrolysis of alkyl thiochloroformates was for the operation of a dominant S_N_1 mechanism. This proposition was based on observations of studies of the hydrolysis of various alkyl thiochloroformates, chloroformates, and carbamoyl chlorides, including determination of their hydrolysis activation parameters and the solvent deuterium isotope effects. Lee *et al*. [61,62] favored an S_N_1 mechanism for the methyl dithiochloroformate ester (MeSCSCl), and competing S_N_1 and S_N_2 pathways for methyl chlorothioformate, (MeSCOCl), and methyl chlorothionoformate, (MeOCSCl), with the extent of operation of each mechanism being dependent on the proportion of water in the aqueous solvents. Castro, Santos and co-workers [63–68] advanced a stepwise mechanism through a zwitterionic tetrahedral intermediate for a variety of thio-, dithio-, and thiono- analogs of carboxylic acids.

In the present paper, we report the first-order specific rate constants at 25.0 °C for the solvolyses of **1** in ethanol and methanol and thirteen binary mixtures of aqueous ethanol (EtOH), aqueous methanol (MeOH), aqueous acetone, aqueous 2,2,2-trifluoroethanol (TFE), and aqueous 1,1,1,3,3,3-hexafluoro-2-propanol (HFIP). Studies were also carried out in five mixtures of TFE and EtOH. Rate constants at additional temperatures and the calculated activation parameters are also reported.

## 2. Results and Discussion

As reported in Table 1, the first-order specific rate constants for **1** increase as the amount of water in the binary organic mixtures increases. This upward rise in the rate coefficients coupled with the incremental water content is also observed in the highly ionizing aqueous fluoroalcohol mixtures. These observations are consistent with an ionization mechanism (S_N_1) with some nucleophilic participation of the solvent in the transition state. In Table 1, we report the rate data for **1** in twenty solvents together with the literature values for *N*_T_ [37,38] and *Y*_Cl_ [34–36]. Additionally for **1**, rates were measured at 35.0 °C and 45.0 °C for 100% MeOH, 100% EtOH, 80% EtOH, and 90% acetone, and at 15.0 °C and 20.0 °C for 97% TFE, 70% TFE, 90% HFIP, and 70% HFIP. Included in the footnotes of Table 1, are the derived Arrhenius parameters (ΔH^≠^ and ΔS^≠^) at 25.0 °C for 100% MeOH, 100% EtOH, 80% EtOH, 90% acetone, 97% TFE, 90% TFE, 90% HFIP, and 70% HFIP. Also reported are the specific rate ratios for **1** and **2** and previously tabulated ratios for the corresponding ethyl [10] and phenyl [26] esters.

**Scheme 2.**
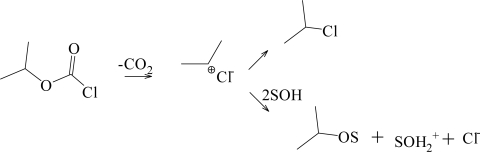
Solvolysis-decomposition of isopropyl chloroformate (**2**).

In Table 2, the Grunwald-Winstein *l*, m, and *c* values, the goodness-of-fit, and *F*-test parameters for a variety of ROCOCl, RSCSCl, ROCSCl, and RSCOCl solvolyses are reported. The *l* and *m* values obtained for MeOCOCl are very similar to those obtained for PhOCOCl, hence it was suggested [12] that, due to the strong electron-withdrawing ability of the methoxy group, MeOCOCl solvolyses by a dominant bimolecular addition-elimination mechanism in all solvents except 90% HFIP. In MeSCOCl, the mesomeric effect of the thiomethoxy group predominates in the highly ionizing aqueous fluoroalcohols and 60% acetone resulting in a unimolecular mechanism [30] with a profound rear-side nucleophilic solvation of the developing carbocation. In the more nucleophilic solvents, the mechanism switches to a bimolecular addition-elimination with the addition step being rate determining. It was also shown [30] that there was a tendency for both reaction channels to operate simultaneously in MeSCOCl. Increasing the chain length by a single carbon in ethyl chloroformate, (EtOCOCl), diminishes the alkoxy group’s inductive ability slightly, and dual reaction channels [10] are followed, with an addition-elimination in the more nucleophilic solvents and a S_N_1 pathway with appreciable nucleophilic solvation of the developing acylium ion, in the aqueous fluoroalcohols. In ethyl chlorothioformate, (EtSCOCl), the relative importance of the two reaction channels is reversed [10], where the unimolecular ionization pathway is followed in a majority of the solvents and an addition-elimination channel in methanol, ethanol, and 90% ethanol. The influence of a branch-chain alkyl group as in isopropyl chloroformate, (**2**, *i-*PrOCOCl) [24], induces a counterbalance between the hyperconjugative mesomeric effect and the inductive ability of the alkyl group, resulting in a bimolecular addition-elimination pathway in the more nucleophilic solvents and a unimolecular fragmentation-ionization pathway in which ionization is accompanied by loss of carbon dioxide, in the majority of the solvents studied (Scheme 2).

A correlation using all twenty data points listed in Table 1 within the simple Grunwald-Winstein equation (Equation 1) resulted in a rather poor correlation coefficient *R* = 0.930, *m* value of 0.43 ± 0.04, *c* value of −0.44 ± 0.09, and a *F*-test value of 114. The same set of solvents using Equation 2 yields, *l* = 0.30 ± 0.13, *m* = 0.63 ± 0.09, *c* = −0.26 ± 0.11, *R* = 0.947, and a *F*-test value of 74. Using Equation 1 and omitting the rate constant in 100 EtOH results in, *m* = 0.45 ± 0.04, *c* = −0.49 ± 0.10, *R* = 0.933, and *F*-test = 115. For the identical 19 solvents, using Equation 2, we obtained a much improved correlation coefficient *R* = 0.961, *l* = 0.38 ± 0.11, *m* = 0.72 ± 0.09, *c* = −0.28 ± 0.10, and an *F*-test value of 97. The improvements observed using Equation 2, in the correlation coefficient and the *F*-test values on omission of the 100 EtOH data point, is a strong indicator that there is a superimposed bimolecular pathway in this solvent. Utilizing the equation 0.38 *N*_T_ + 0.72 *Y*_Cl_ −0.28 for log (*k*/*k*_o_), we estimate the ionization rate (*k*) in 100% EtOH to be, 0.158 × 10^−5^ s^−1^. This value indicates that, in 100% ethanol, 87% of the reaction is occurring by the bimolecular channel. For 90% EtOH and 90% acetone, the corresponding values are 44% and 34% respectively.

**Figure 2.**
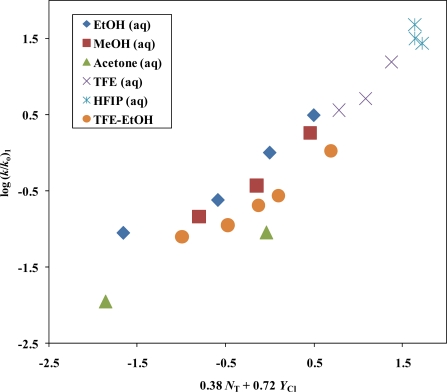
The plot of log (*k/k_o_*) for isopropyl chlorothioformate (**1**) against 0.38 *N*_T_ + 0.72 *Y*_Cl_ in the twenty common pure and binary solvents. The point for the 100% EtOH was not included in the correlation. It is added to show the extent of its deviation.

With use of Equation 2, the *l* and *m* values for solvolyses of **1** imply that the stepwise ionization pathway is accompanied by a modest rear-side nucleophilic solvation of the developing S_N_1 transition state. As in the case of MeSCOCl [30], the entropies of activation (ΔS^≠^ values) for **1** reported in the footnotes of Table 1, are negative. The negative ΔS^≠^ values suggest a greater ordering of the solvent molecules in the transition state, encasing the developing carbocation and thus dispersing the electrical charge. From the data in Table 2, the observed trend for the *l* values as one progresses with the increasing carbon chain length, requires a necessary decrease in nucleophilic solvation requirements as one moves from MeSCOCl to **1**, due to an increase in the hyperconjugative mesomeric effects. The *l* value of 0.79 ± 0.06 and m value of 0.85 ± 0.07 obtained for MeSCOCl [30] are similar to the *l* value of 0.83 ± 0.06, and *m* = 0.70 ± 0.04 observed for the solvolysis of acetyl chloride [69], whereas the *l* value of 0.38 ± 0.11 and *m* value of 0.72 ± 0.09 obtained for **1**, are similar to *l* = 0.34 ± 0.04 and *m* = 0.84 ± 0.02 obtained for *t*-butyl chloride [35,45]. A plot of log (*k*/*k*_o_)_**1**_ *versus* 0.38 *N*_T_ + 0.72 *Y*_Cl_ as shown in Figure 2, is consistent with the previous discussion of the side-by-side operation of the addition-elimination and stepwise ionization (with nucleophilic solvation) mechanism in a few of the more nucleophilic solvents.

**Scheme 3.**
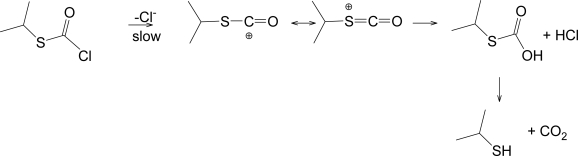
Unimolecular step-wise hydrolysis of isopropyl chlorothioformate (1).

Queen *et al*. [59] found 2-propanethiol as a product of the hydrolysis of 1. Since they found no appreciable amounts of isopropyl alcohol, propene, or carbonyl sulfide, they suggested that there was no alkyl-sulfur bond fission in the hydrolysis of **1**. These observations are consistent with the results outlined above and with the mechanistic scheme (Scheme 3) shown below.

The very different responses of the specific rate ratios for RSCOCl/ROCOCl to solvent variation depending on whether the R group is phenyl, ethyl, or isopropyl (Table 1) are nicely consistent with the conclusions drawn from the application of the extended Grunwald-Winstein equation (Equation 2). When R = Ph, the very low values (0.026–0.042) for solvolyses in the non-fluoroalcohol-containing solvents are consistent with the proposed addition-elimination mechanism, with the carbonyl carbon more prone to the rate-determining addition when the better electron-withdrawing phenoxy group is attached. In solvents with high fluoroalcohol content the ratio increases in value and reaches values of 5.9 for 97% TFE and 16 for 90% HFIP, where a superimposed S_N_1 mechanism becomes dominant for PhSCOCl solvolyses.

A different pattern of behavior is observed [10] for R = Et (Table 1), with ratios varying from 0.19 (100% ethanol) to 357 (90% HFIP). Both reaction channels are observed with the unimolecular (S_N_1) covering the larger range of solvent composition for EtSCOCl and the addition-elimination the larger range of solvent composition for EtOCOCl. The small (0.19 to 0.40) values in MeOH and EtOH and in MeOH-H_2_O and EtOH-H_2_O mixtures can be considered to reflect the dominance of the addition-elimination mechanism and the much larger (89–357) values in TFE-H_2_O and HFIP-H_2_O mixtures the dominance of the ionization (S_N_1) mechanism for both substrates.

The values for the *i*-PrSCOCl/*i-*PrOCOCl specific rate ratios behave in yet a different manner. There is very little variation in the specific rate ratio over the full range of solvents considered when the presently determined values for **1** are compared with the values reported earlier [24] for **2**. Since both show a moderate *l* value and a fairly high *m* value over the more ionizing solvents, it would appear that both are reacting by an ionization mechanism. In this case, the values of in excess of over 100 when ionization is believed to be followed for the ethyl derivatives might be expected to be applicable also to the isopropyl derivatives. In reality, the ratio (Table 1) only reaches a value of just over ten. We tentatively propose that this is a consequence of the rate-determining step producing the carboxylium ion for **1** (Scheme 3). This view is supported by the observation of Queen and coworkers [59] of 2-propanethiol as the major product for the hydrolyses of **1**, an observation that requires the retention of the isopropyl-sulfur bond throughout the solvolysis process. This process is probably a consequence of the sulfur being able to support appreciable positive charge in the resonance-stabilized carboxylium ion. If the corresponding process operated for the carboxylium ion from **2**, the positive charge would be partially transferred to the oxygen (oxonium ion character). This would lead to the electronegative oxygen exerting a strong pull on the electrons of the isopropyl-oxygen bond which is apparently sufficient for the mechanism of the rate-determining step to be best described as in Scheme 2, with a rate-determining ionization-fragmentation process. To dominate, this process must be favored relative to the alternative process producing the carboxylium ion from **2** (parallel to the process of Scheme 3). This leads to a reduction the *i-*PrSCOCl/*i-*PrOCOCl ratio relative to that predicted if both proceeded with a carboxylium ion formation as the rate-determining step.

## 3. Conclusions

It is becoming clear that the solvolyses of alkyl chlorothioformates involve simultaneous side-by-side reaction channels where the mesomeric effect governs the dominant mechanism, which varies with the ionizing ability of the solvent. For the solvolyses of isopropyl chlorothioformate (**1**) in all solvents but 100% EtOH, we propose a dominant stepwise S_N_1 mechanism with discernible rear-side nucleophilic solvation of the resonance stabilized acylium ion (Scheme 3). Conversely in isopropyl chloroformate (**2**), the inductive pull of the isopropoxy group is paramount, and dual distinct channels are observed, with a bimolecular addition-elimination in the more nucleophilic solvents and a unimolecular fragmentation-ionization mechanism (Scheme 2) in the highly ionizing solvents. The extended Grunwald-Winstein equation was able to differentiate between such mechanisms and it continues to play a major role in studies using linear free energy relationships.

The very different responses of the RSCOCl/ROCOCl ratio to changes in the R group are nicely consistent with a predominantly addition-elimination process when R is Ph, perturbed for highly ionizing solvents by the incursion of an ionization component for the PhSCOCl solvolyses. When R is Et, both reaction channels are observed for each substrate but with the unimolecular dominant for EtSCOCl and the addition-elimination for EtOCOCl. The lowest values for the ratio are for the addition-elimination pathway and the highest values for the ionization pathway. Similar low values are observed when R is *i-*Pr but with the range where the addition-elimination mechanism is dominant being very limited for *i-*PrSCOCl. At first sight, a surprising aspect is that, in the highly ionizing solvents, the ratio has a much lower value than when R = Et. This is rationalized in terms of the *i-*PrOCOCl involving a favored ionization-fragmentation process, which must therefore be faster than what the carboxylium ion forming process would be, with a reduction in the value of the *k*_**1**_/*k*_**2**_ ratio, relative to the corresponding value when R = Et. This rationalization is nicely consistent with the observation that the *s*-isopropyl thioesters have a higher torsional barrier when compared to the *s*-ethyl thioesters [55].

## 4. Experimental Section

The isopropyl chlorothioformate (96%, Sigma-Aldrich) was used as received. Solvents were purified and the kinetic runs carried out as described previously [10]. A substrate concentration of approximately 0.005 M in a variety of solvents was employed. For some of the runs, calculation of the specific rates of solvolysis (first-order rate coefficients) was carried out by a process in which the conventional Guggenheim treatment [70] was modified [71] so as to give an estimate of the infinity titer, which was then used to calculate for each run a series of integrated rate coefficients. The specific rates and associated standard deviations, as presented in Table 1, are obtained by averaging all of the values from, at least, duplicate runs.

Multiple regression analyses were carried out using the Excel 2007 package from the Microsoft Corporation, and the SigmaPlot 9.0 software version from Systat Software, Inc., San Jose, CA, was used for the Guggenheim treatments.

## Figures and Tables

**Table 1.** Specific rates of solvolysis (*k*) of 1, in several binary solvents at 25.0 °C, literature values for *N_T_* and *Y_Cl_*, specific rate ratios for 1 and 2, and the corresponding ratios for the ethyl and phenyl compounds.

**Solvent (%)*a***	**1 @ 25.0 °C; 10^5^*k,* s^−1^*b***	***N_T_^c^***	***Y_Cl_^d^***	***k*_1_/*k*_2_^e^**	**Et*f***	**Ph*g***
100% MeOH	1.99 ± 0.11*h*	0.17	−1.2	0.47	0.26	0.042
90% MeOH	5.06 ± 0.24	−0.01	−0.20	0.61		
80% MeOH	24.7 ± 0.3	−0.06	0.67	1.84	0.40	
100% EtOH	1.21 ± 0.06*i*	0.37	−2.50	1.11	0.19	0.026
90% EtOH	3.32 ± 0.17	0.16	−0.90	1.41	0.21	0.029
80% EtOH	13.7 ± 0.7*j*	0.00	0.00	3.49	0.37	0.029
70% EtOH	42.1 ± 1.3	−0.20	0.80	7.61		0.029
90% Acetone	0.153 ± 0.004*k*	−0.35	−2.39	0.46		
70% Acetone	1.21 ± 0.06	−0.42	0.17	0.47		
97% TFE (w/w)	49.8 ± 2.5*l*	−3.30	2.83	4.05	260	5.9
90% TFE (w/w)	69.5 ± 2.1	−2.55	2.85	5.00	171	0.50
70% TFE (w/w)	212 ± 18*m*	−1.98	2.96	10.76	89	
80T-20E	14.5 ± 0.8	−1.76	1.89	4.45		0.10
60T-40E	3.75 ± 0.18	−0.94	0.63	2.66		0.033
50T-50E	2.81 ± 0.02	−0.64	0.60			
40T-60E	1.55 ± 0.16	−0.34	−0.48	1.61		
20T-80E	1.09 ± 0.13	0.08	−1.42	1.44		
97%HFIP (w/w)	376 ± 17	−5.26	5.17	2.58	253	
90%HFIP (w/w)	437 ± 28*n*	−3.84	4.41	6.91	357	16
70%HFIP (w/w)	659 ± 39*o*	−2.94	3.83	10.97	183	0.22*p*

a Substrate concentration of *ca.* 0.0052 M; binary solvents on a volume-volume basis at 25.0 °C, except for TFE-H_2_O and HFIP-H_2_O solvents which are on a weight-weight basis. T-E are TFE-ethanol mixtures.

b With associated standard deviation.

c Values taken from [37,38].

d Values taken from [34–36].

e The *k*_**2**_ values from ref. 24.

f Values for *k*_(EtSCOCl)_/*k*_(EtOCOCl)_ from [10].

g Values for *k*_(PhSCOCl)_/*k*_(PhOCOCl)_ from [26].

h A value of 6.00 (± 0.30) × 10^−5^ s^−1^ and a value of 19.3 (± 0.15) × 10^−5^ s^−1^ was obtained at 35.0 °C and 45.0 °C respectively. ΔH^≠^ = 20.8 ± 0.7 kcal/mol, ΔS^≠^ = −10.4 ± 8 cal mol^−1^ K^−1^.

i A value of 2.34 (± 0.19) × 10^−5^ s^−1^ and a value of 6.11 (± 0.17) × 10^−5^ s^−1^ was obtained at 35.0 °C and 45.0 °C respectively. ΔH^≠^ = 14.6 ± 1.9 kcal/mol, ΔS^≠^ = −32.1 ± 6.5 cal mol^−1^ K^−1^.

j A value of 21.0 (± 0.11) × 10^−5^ s^−1^ and a value of 42.4 (± 0.18) × 10^−5^ s^−1^ was obtained at 35.0 °C and 45.0 °C respectively. ΔH^≠^ = 10.4 ± 1.4 kcal/mol, ΔS^≠^ = −41.4 ± 4.9 cal mol^−1^ K^−1^.

k A value of 0.248 (± 0.020) × 10^−5^ s^−1^ and a value of 0.513 (± 0.030) × 10^−5^ s^−1^ was obtained at 35.0 °C and 45.0 °C respectively. ΔH^≠^ = 10.8 ± 1.6 kcal/mol, ΔS^≠^ = −49.0 ± 5.7 cal mol^−1^ K^−1^.

l A value of 14.7 (± 0.1) × 10^−5^ s^−1^ and a value of 26.7 (± 0.2) × 10^−5^ s^−1^ was obtained at 15.0 °C and 20.0 °C respectively. ΔH^≠^ = 20.2 ± 0.5 kcal/mol, ΔS^≠^ = −5.8 ± 2.0 cal mol^−1^ K^−1^.

m A value of 81.4 (± 3.6) × 10^−5^ s^−1^ and a value of 145 (± 9) × 10^−5^ s^−1^ was obtained at 15.0 °C and 20.0 °C respectively. ΔH^≠^ = 15.8 ± 1.8 kcal/mol, ΔS^≠^ = −17.8 ± 6.3 cal mol^−1^ K^−1^.

n A value of 162 (± 8) × 10^−5^ s^−1^ and a value of 244 (± 11) × 10^−5^ s^−1^ was obtained at 15.0 °C and 20.0 °C respectively. ΔH^≠^ = 16.3 ± 1.9 kcal/mol, ΔS^≠^ = −14.6 ± 6.8 cal mol^−1^ K^−1^.

o A value of 212 (± 12) × 10^−5^ s^−1^ and a value of 358 (± 19) × 10^−5^ s^−1^ was obtained at 15.0 °C and 20.0 °C respectively. ΔH^≠^ = 18.8 ± 1.0 kcal/mol, ΔS^≠^ = −5.6 ± 4.2 cal mol^−1^ K^−1^.

p For 50% HFIP (w/w).

**Table 2.** Correlation of the specific rates of solvolysis of a variety of ROCOCl, RSCOCl, RSCSCl, and ROCSCl substrates using the extended Grunwald-Winstein equation (Equation 2).

**Substrate**	***n^a^***	***l^b^***	***m^b^***	***c^c^***	***R^d^***	***F^e^***	***Mechanism***
PhOCOCl*f*	49	1.66 ± 0.05	0.56 ± 0.03	0.15 ± 0.07	0.980	568	A-E
PhSCSCl*g*	31	0.69 ± 0.05	0.95 ± 0.03	0.18 ± 0.05	0.987	521	S_N_1
PhOCSCl*h*	9	1.88 ± 0.28	0.56 ± 0.15	0.38 ± 0.15	0.950	28	A-E
	18	0.34 ± 0.05	0.93 ± 0.09	−2.54 ± 0.34	0.955	77	S_N_1
PhSCOCl*i*	16	1.74 ± 0.17	0.48 ± 0.07	0.19 ± 0.23	0.946	55	A-E
	6	0.62 ± 0.08	0.92 ± 0.11	−2.29 ± 0.13	0.983	44	S_N_1
EtOCOCl*j*	28	1.56 ± 0.09	0.55 ± 0.03	0.19 ± 0.24	0.967	179	A-E
	7	0.69 ± 0.13	0.82 ± 0.16	−2.40 ± 0.27	0.946	17	S_N_1
EtSCOCl*k*	19	0.66 ± 0.08	0.93 ± 0.07	−0.16 ± 0.31	0.961	96	S_N_1
MeOCOCl*l*	19	1.59 ± 0.09	0.58 ± 0.05	0.16 ± 0.07	0.977		A-E
MeSCOCl*m*	12	1.48 ± 0.18	0.44 ± 0.06	0.08 ± 0.08	0.949	40	A-E
	8	0.79 ± 0.06	0.85 ± 0.07	−0.27 ± 0.18	0.987	95	S_N_1
*i-*PrOCOCl*n*	9	1.35 ± 0.22	0.40 ± 0.05	0.18 ± 0.07	0.960	35	A-E
	16	0.28 ± 0.04	0.59 ± 0.04	−0.32 ± 0.06	0.982	176	fragmentation-ionization
*i*-PrSCOCl*o*	19	0.38 ± 0.11	0.72 ± 0.09	−0.28 ± 0.10	0.961	97	S_N_1

a *n* is the number of solvents.

b With associated standard error.

c The earlier values are accompanied by standard error of the estimate.

d Correlation coefficient.

e *F*-test value.

f Values taken from [21].

g Values taken from [21].

h Values taken from [21].

i Values taken from [21].

j Values taken from [10].

k Values taken from [10].

l Values taken from [12].

m Values taken from [30].

n Values taken from [24].

o This work without 100% EtOH.
